# Identifying Aspirin as a Contributing Factor to Persistent Gout: A Case Report

**DOI:** 10.7759/cureus.85940

**Published:** 2025-06-13

**Authors:** Justin Nguyen, Yao Liang, Abir Islam, Venu Kakarala, Matthew D Overturf

**Affiliations:** 1 Medicine, Edward Via College of Osteopathic Medicine, Monroe, USA; 2 Outpatient Internal Medicine, Zachary Internal Medicine, Slaughter, USA; 3 Anatomical Sciences, Edward Via College of Osteopathic Medicine, Monroe, USA

**Keywords:** clopidogrel, gout flare, low dose aspirin, side effects of medical treatment, uric acid levels

## Abstract

Gout is a metabolic disorder characterized by acute and chronic inflammatory responses due to monosodium urate (MSU) crystal deposition, commonly affecting the first metatarsophalangeal joint, hands, and/or knees. While this condition is typically self-limiting, recurrent cases can suggest an underlying contributor. Low-dose aspirin is commonly used as a cardiovascular prophylaxis. Although research is limited, aspirin has been implicated in decreased uric acid excretion, thus potentially exacerbating gout symptoms in patients. This case highlights a 67-year-old African American male who presented to a clinic complaining of gout in his right hand. Despite following strict lifestyle practices and medication compliance, pharmacological therapies were rendered ineffective until aspirin was identified as a contributing factor to his gout. Optimizing his medication regimen resolved his gout attacks, which stresses the concept that clinicians must be diligent with lesser-known side effects of medication to improve patient outcomes.

## Introduction

Gout is a metabolic disease that causes acute and/or chronic inflammatory responses through activation of the innate immune system due to the accumulation of monosodium urate (MSU) crystals. This buildup can lead to pain, swelling, and stiffness in the affected joint, most commonly the first metatarsophalangeal joint. In most cases, urate underexcretion through renal and gut metabolism is the main cause of gout [1]. The high levels of MSU crystals trigger an inflammatory response of the body, modulated by IL-1B [1-3]. This interleukin upregulates cytokines and chemokines, resulting in the recruitment of neutrophils [1,2]. These neutrophils degrade cytokines and chemokines through proteases, promoting resolution of the inflammation [2,3]. In severe cases, advanced gout is typically characterized by structural damage and/or development of tophi, which are chronic foreign body structures that contain MSU crystals surrounded by inflammatory cells and connective tissue [2,3].

There are several risk factors for gout, including, but not limited to, obesity, hypertension (HTN), chronic kidney disease (CKD), diabetes mellitus (DM), hyperlipidemia (HLD), and metabolic syndrome (in a table in the Discussion section) [3]. A gout diagnosis is associated with increased risk of ischemic heart disease and cerebrovascular disease/events [4]. Treatment options for acute and/or chronic gout include lifestyle modifications, symptomatic and preventative medications, which are also indicated for this disease process. In most cases, the disease process is typically self-limiting and resolves within a few days or weeks; however, for acute symptomatic treatment, therapies such as NSAIDs, glucocorticoids, and/or colchicine are recommended [4]. For chronic gout, prophylactic pharmacological agents such as allopurinol, febuxostat, or uricosuric drugs can have a positive effect [4]. Regardless, treatment and care should always be individualized and tailored to patient needs.

We present a case of a 67-year-old male who presented to our rural medicine clinic with the diagnosis of gout that was resistant to traditional treatment modalities due to a lesser-known side effect of his medication regimen.

## Case presentation

A 67-year-old non-obese African American male presented to the internal medicine clinic for an evaluation of severe right-hand pain and swelling. The onset of the pain was three to four days prior to his new patient visit. The patient described the pain as a sharp, constant, non-radiating sensation exacerbated by wrist movement. The patient reported that he had experienced similar episodes in the past and had been using colchicine inconsistently; however, it did not provide relief, and therefore, he had stopped taking the medication.

His past medical history included HTN, dyslipidemia, gout, gastroesophageal reflux disease (GERD), CKD stage 3, and a significant family history of cardiovascular disease. His body mass index (BMI) was noted to be 24.78 kg/m2. At the time of his visit, his reported medications included daily 81 mg of aspirin, 0.6 mg of colchicine, and 90 mg of nifedipine extended release (ER). Socially, he denied illicit drug use, smoking, or drinking alcohol. He reported a balanced diet, limiting red meat intake, and adhering to a low-purine diet to manage his gout flares.

On physical exam, his right hand exhibited tenderness and swelling, and pain was elicited with joint manipulation. Based on his medical history and physical exam, the initial differential diagnosis included gout, osteoarthritis, and trauma-induced joint effusion. Uric acid levels were elevated at 8.3 mg/dL, and X-rays of the hand were within normal limits. A gout diagnosis was made, and treatment included restarting allopurinol 100 mg, NSAIDs, and 60 mg of ketorolac for symptom relief.

Two weeks later, the patient contacted the clinic with more gout flare-ups. Uric acid levels were retested at 5.3 mg/dL, which were within normal limits. Therefore, 40 mg of febuxostat was prescribed in place of allopurinol. One week passed, and the patient still complained of persistent symptoms despite these adjustments. At that time, uric acid levels were tested again, which showed a level of 3.0 mg/dL. Table 1 illustrates the trend of uric acid throughout the patient's visits, along with clinical notes. The patient reported medication regimen compliance. He denied drinking any alcohol or making any new additions to his diet.

**Table 1:** Patient's uric acid level responses to medication changes D - Day; H - High; L - Low

Day	Uric Acid (mg/dL)	Reference	Clinical Notes
D0	8.3 (H)	4.0-8.0 mg/dL	Initial visit. Uric acid elevated. Prescribed allopurinol.
D14	5.3	4.0-8.0 mg/dL	Follow-up visit for persistent gout symptoms despite normal uric acid levels. Substituted allopurinol for febuxostat.
D21	3.0 (L)	4.0-8.0 mg/dL	Follow-up visit for persistent gout after medication changes. Opted to change low-dose aspirin to clopidogrel.

Upon medication reconciliation, it was noted that he was on a daily regimen of low-dose aspirin. Given the limited but potential impact of aspirin on uric acid retention, clopidogrel was chosen as a substitute for aspirin. Following this change, the patient’s symptoms resolved within a few days. Since this substitution, the patient has not had any gout flares since starting clopidogrel and continues to remain symptom-free.

## Discussion

The patient presented with severe pain and swelling in the right hand, along with elevated serum uric acid levels, consistent with a gout flare. Colchicine, followed by the addition of allopurinol and NSAIDs, did not help alleviate the patient’s symptoms. A subsequent switch to febuxostat in combination with dual therapies also failed to alleviate symptoms. The persistence of symptoms, despite achieving target uric acid levels and appropriate gout management, prompted further investigation into potential contributing factors.

Risk factors are important to consider in determining the diagnosis of gout (Table 2). African Americans have a twofold increased risk of gout compared to Caucasians, with a respective incidence rate of 3.11 and 1.82 per 1,000 person-years, respectively, which can be partially attributed to the increased incidence of HTN in African Americans [5]. Additionally, patients with moderate-to-severe CKD have a higher risk of developing severe gout and/or tophaceous deposits [6]. The patient’s HTN may have increased the likelihood of developing gout. One of the first-line therapies for essential HTN is thiazide diuretics according to the Eighth Joint National Committee (JNC-8) guidelines; however, thiazide diuretics have a side effect profile of increased uric acid reabsorption [7,8]. While the patient was initially on this medication in the past, he was switched to a calcium channel blocker, nifedipine.

**Table 2:** Gout risk factors [3,5] Bold texts indicate a positive risk factor. ACE - Angiotensin-Converting Enzyme; ARB - Angiotensin II Receptor Blocker; CHF - Congestive Heart Failure; CKD - Chronic Kidney Disease; HTN - Hypertension

Risk Factor	Example
Genetics and age	Male sex, ancestry, increasing age, menopause
Underlying medical problems	Obesity, weight changes, CKD, HTN, dyslipidemia, CHF, obstructive sleep apnea, anemia, psoriasis, sickle cell anemia, malignancy, lead exposure
Medications	Diuretics, cyclosporin, tacrolimus, ACE inhibitors, ARBs, beta blockers, pyrazinamide, ritonavir
Diet	Red meat, seafood, alcohol

**Figure 1:**
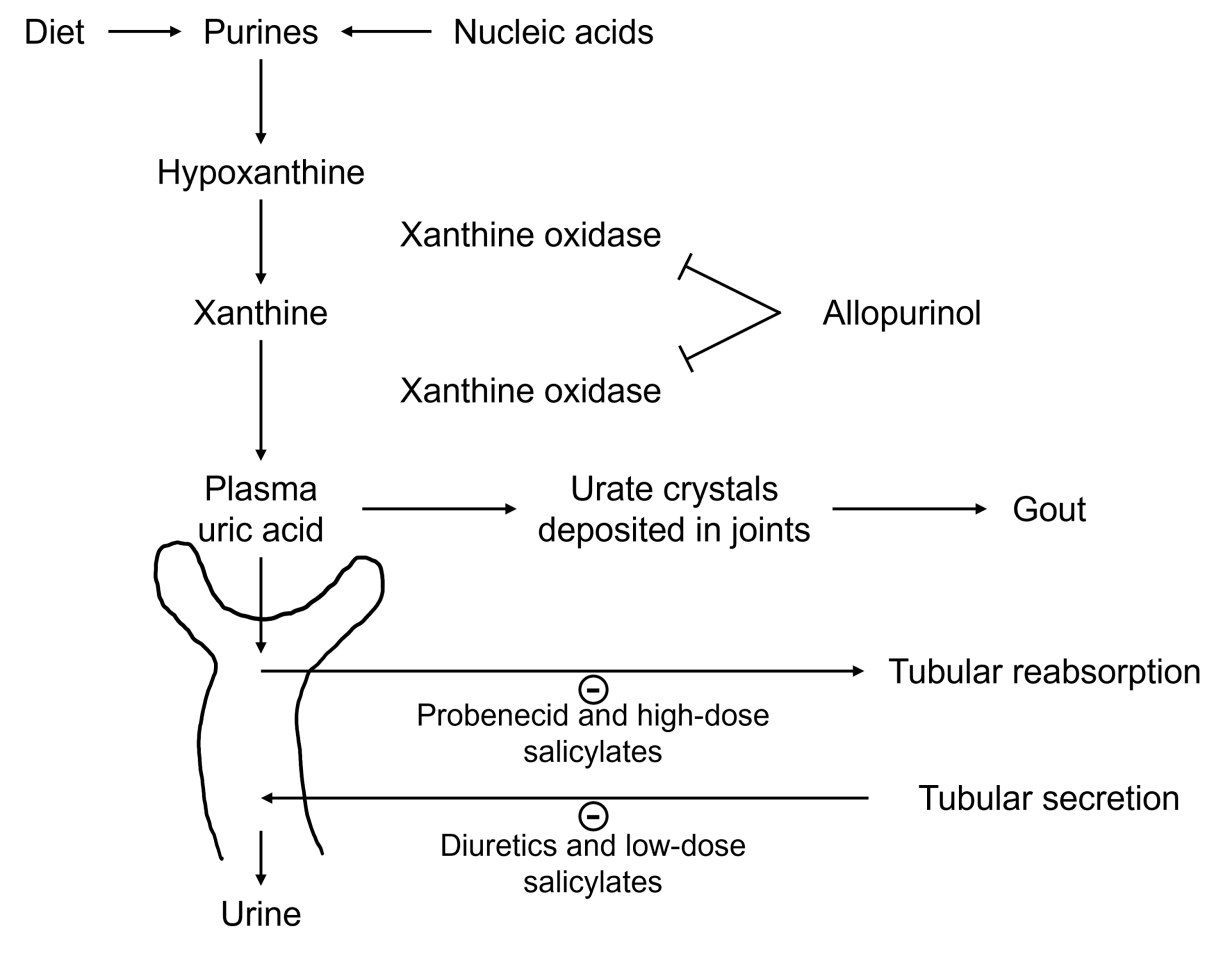
Purine degradation pathway High-dose salicylates prevent tubular reabsorption of uric acid. Low-dose salicylates prevent tubular secretion of uric acid. Adapted from Medbullets [12].

Although aspirin is not a primary indication for patients with CKD, it is commonly used for its antiplatelet effects, especially in patients with other cardiovascular risk factors, consistent with this patient’s significant family history of heart disease [9,10]. However, at low doses (<325 mg/day), aspirin may inhibit the renal excretion of uric acid via the purine degradation pathway, potentially exacerbating hyperuricemia and triggering recurrent gout (Figure 1) [11]. The association is decreased with increased aspirin dosages; however, the literature is unclear, as some sources cite caution with the use of high-dose aspirin in patients with gout, pointing to a discrepancy that needs further research [11]. It is suspected that this patient’s daily low-dose aspirin regimen contributed to the persistence of gout symptoms despite a normal range of serum uric acid levels.

**Figure 2:**
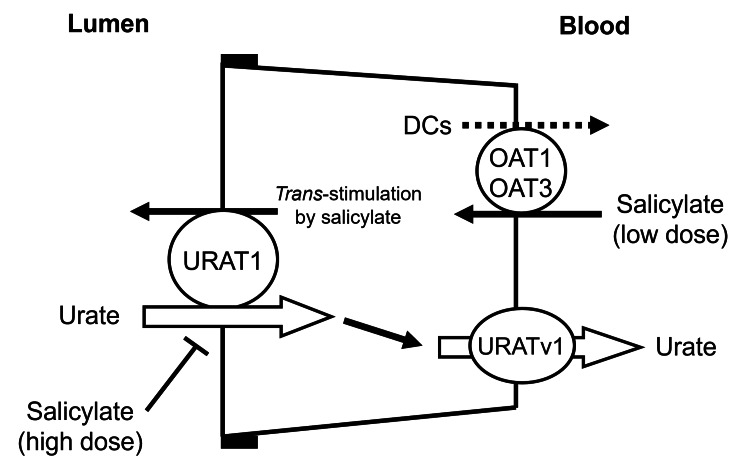
Urate transporter (URAT1)-mediated transport of uric acid and salicylates The image depicts the proposed mechanism of high and low dose salicylates’ role in uric acid. At low doses, aspirin acts as an exchange substrate and facilitates urate reabsorption, whereas at high doses it acts as an inhibitor of urate reabsorption. Adapted from Ohtsu et al. [13].

Although the effects of aspirin and uric acid levels are documented in a few studies, it is still considered a side effect that can be overlooked. There are several proposed mechanisms for this uric acid retention. In a proposed model by Ohtsu et al., low-dose salicylates (60-300 mg) act as an exchange substrate with dicarboxylates (DCs) via organic anion transporters (OAT1/OAT3) and facilitate urate reabsorption through the urate transporter 1 (URAT1) of the nephrogenic apical lumen (Figure 2) [13]. This result increases blood uric acid levels, which can potentially lead to gout. Whereas at higher doses, aspirin acts as an inhibitor of urate reabsorption, which may prevent uric acid accumulation in the blood and may serve as a protective property against gout [13]. Other models propose that some uricosuric drugs and salicylates have a bimodal action on urate renal excretion [13,14]. Although the mechanisms of action are not clear, researchers have identified that a urate efflux transporter, multidrug resistance protein 4 (MRP4), is inhibited by salicylates, which can also increase uric acid levels [14].

In this case, substituting aspirin with clopidogrel was based on limited data that aspirin's effect on uric acid retention was contributing to the patient's recurrent gout flares. However, it is important to note that elevated uric acid levels are not always indicative of gout [2]. Clopidogrel, which does not affect uric acid levels, was chosen as an alternative antiplatelet therapy. Clinicians may use online external resources to help guide them with medical decision-making in accordance with updated and recommended guidelines [15]. In patients with significant cardiovascular comorbidities and risk factors, the use of low-dose aspirin should be encouraged based on current guidelines set forth by the US Preventive Services Task Force (USPSTF) [9]. While the USPSTF concludes with moderate certainty that initiating aspirin for primary prevention of cardiovascular events in adults aged 60 years or older yields no net benefit, this patient has been taking aspirin chronically due to his risk factors [9]. Moreover, this patient's age is considered one of the strongest risk factors for cardiovascular disease, thus supporting continued use of antiplatelet therapy [9]. The decision to place the patient on clopidogrel was made because of its cardioprotective properties, much like aspirin.

Although this clopidogrel substitution may seem like a straightforward plan, one must consider costs. According to a cost-effectiveness analysis conducted in a South Korean healthcare system, clopidogrel monotherapy costs $3,192 more in lifetime healthcare costs compared to aspirin [16]. A single 75 mg of clopidrogel tablet costs around $3.22 versus $0.04 for a single 325 mg aspirin tablet [17]. In any case, a cost-benefit analysis should be used and tailored to individual needs, especially for this patient experiencing persistent gout attacks. While this monotherapy may be more costly, following this switch, the patient's gout symptoms resolved, and no further flare-ups were reported.

## Conclusions

This case highlights the importance of recognizing a lesser-known side effect of aspirin: its potential to increase uric acid levels. Popular clinical websites, such as Epocrates and UpToDate, are widely used, valuable resources for accessing drug information, interactions, dosing guidelines, and treatment protocols. Neither resource fully addresses this side effect in the appropriate context. The omission of elevated uric acid levels from the aspirin side effect profile can inadvertently impact patient care and treatment optimization. In the case of this patient, aspirin delayed care and prolonged his symptoms and suffering. Therefore, it is imperative for healthcare providers to be aware of this effect to ensure more comprehensive patient management and better therapeutic outcomes.
